# Molecular classification of muscle-invasive bladder cancer based on a simplified immunohistochemical panel using GATA3, CK5/6, and p16

**DOI:** 10.17305/bb.2023.9242

**Published:** 2023-12-01

**Authors:** Robert Terlević, Monika Ulamec, Goran Štimac, Jure Murgić, Božo Krušlin

**Affiliations:** 1 Department of Pathology and Cytology, Pula General Hospital, Pula, Croatia; 2 Clinical Department of Pathology and Cytology “Ljudevit Jurak”, Clinical Hospital Center Sestre milosrdnice, Zagreb, Croatia; 3 School of Medicine, University of Zagreb, Zagreb, Croatia; 4 Clinical Department of Urology, Clinical Hospital Center Sestre milosrdnice, Zagreb, Croatia; 5 Clinical Department of Oncology, Clinical Hospital Center Sestre milosrdnice, Zagreb, Croatia

**Keywords:** Urothelial carcinoma, bladder cancer, immunohistochemistry (IHC), molecular subtype, survival

## Abstract

The choice of therapy for muscle-invasive bladder cancer (MIBC) could be influenced by the tumor’s molecular subtype. Currently, well-defined consensus subtypes are based on tumor microarray mRNA data. Clearly defined and easy-to-use surrogate molecular subtypes, based on immunohistochemistry (IHC) performed on whole slides, are needed to make subtyping cost-effective and useful in routine work and future research. To aid in the development of a simple immunohistochemical classifier, a retrospective single-center series of 92 cases of localized bladder cancer was identified. Routine IHC for GATA 3 transcription factor (GATA3), cytokeratins 5 and 6 (CK5/6), and p16 was performed on whole tissue blocks containing muscle-invasive disease. Electronic medical records were retrieved and searched for clinical variables, treatment, and survival data. The mean age was 69.6 years, and 73% were males. Conservative treatment was used in 55% of cases, while cystectomy with chemotherapy was used in 45%. GATA3 and CK5/6 expression divided cases into broad luminal and basal subtypes, respectively, while p16 expression was used to subclassify luminal cases into luminal papillary and luminal unstable types according to the consensus molecular classification. When subtyped in this way, GATA3 and CK5/6 negative cases showed worse overall survival. Molecular subtyping of MIBC on whole slides containing muscle-invasive tumor using only three commonly used consensus-based antibodies is a feasible and cost-effective method for detecting subtypes of invasive bladder cancer. Future work combining morphological analysis and IHC is needed to fully translate the consensus molecular classification into a comprehensive, cost-effective subtyping strategy.

## Introduction

Bladder cancer is the seventh most common cause of cancer-related deaths in men [1]. Incidence and mortality vary worldwide, in part due to variable exposure to risk factors, such as smoking, aromatic amines, and schistosomiasis [2–4] and accessibility to diagnosis and treatment [5]. For locally advanced and metastatic disease, the 5-year overall survival rates are 60% and 5%, respectively [6]. Until recently, therapeutic options for advanced urothelial carcinoma were very modest. After initial diagnostic transurethral resection (TUR), radical cystectomy was the treatment of choice for muscle-invasive bladder cancer (MIBC) [5, 7]. Due to the high micrometastatic burden at diagnosis, radical cystectomy is preceded by neoadjuvant chemotherapy in eligible patients or/and followed by adjuvant treatment, which improves overall survival [8]. Due to a high burden of comorbidity in the patient population, bladder-sparing approaches are also used, such as repeat TUR or chemoradiotherapy [9]. Immunotherapy in the neoadjuvant setting has shown promising results but is still limited to clinical trials, partly due to the insufficient availability of biomarkers that could be used to select subgroups of patients who respond to therapy [5, 10–13].

In recent years, several groups, namely, the University of North Carolina, MD Anderson Cancer Center, Lund University, and The Cancer Genome Atlas, released molecular classifications of MIBC based on analysis of tumor transcriptomes using panel genes mRNA assays [14–19]. These groups then developed a consensus molecular classification describing six distinct molecular subgroups, of which the Basal/Squamous (Ba/Sq), Luminal Papillary (LumP), and Luminal Unstable (LumU) constitute the majority of cases [20]. Initial evidence suggests that these molecular subgroups play prognostic and therapeutic roles, such as a better response to neoadjuvant chemotherapy in the luminal non-specified (LumNS) subgroup [21–23], a role for anti-FGFR3 therapy in the luminal subtype [24], a response to immunotherapies in the basal and stroma-rich subtypes [25] and in the LumU, LumNS, and neuroendocrine-like subtypes [23], and a better response to chemoradiotherapy in the luminal unstable and basal subtypes [26]. Additionally, the increased expression of EGFR in the basal subgroup could be used for therapeutic targeting [27]. Although initial data showed a better response to neoadjuvant chemotherapy for the basal subgroup, this view has recently been challenged [28].

Currently, mRNA-based molecular subtyping is not widely used diagnostically because of the relatively high cost and technological complexity associated with this method. Similar to the approach in breast cancer molecular subtyping, immunohistochemical surrogate classification of urothelial carcinoma molecular subtypes could be useful and potentially highly applicable in daily clinical care.

**Table 1:** Parameters used in immunohistochemical analysis

**Antibody**	**Clone**	**Vendor**	**Instrument**	**Dilution**	**Epitope retrieval**	**Incubation**	**Control tissue**
GATA3	L50-823	Cell Marque	BenchMark GX	Pre-diluted	HIER for 48 min at 98 ^∘^C	32 min at 36 ^∘^C	Kidney
CK5/6	D5/16 B4	Ventana/Roche	BenchMark GX	Pre-diluted	HIER for 48 min at 98 ^∘^C	30 min at 36 ^∘^C	Tonsil
p16	E6H4	Ventana/Roche	BenchMark GX	Pre-diluted	HIER for 48 min at 98 ^∘^C	12 min at 36 ^∘^C	Tonsil

HIER: Heat-induced antigen retrieval; CK5/6: Cytokeratins 5 and 6.

The goal of this study was to test the feasibility of classifying bladder cancer into at least three molecular subtypes, namely, LumP, LumU, and Ba/Sq, using immunohistochemistry (IHC) and to analyze whether the molecular subtype is associated with survival when specific treatment was taken into account. This approach could potentially lead to routine subtyping of advanced urothelial carcinoma and personalized therapy selection.

## Materials and methods

### Patients

In this retrospective cross-sectional study, 92 consecutive tumor tissue biopsy samples obtained by TUR of the urinary bladder were analyzed. The biopsies were performed between 1 January 2011 and 31 December 2020 and retrieved from the archives of the Department of Pathology and Cytology “Ljudevit Jurak” of the Clinical Hospital Centre Sestre milosrdnice, Zagreb, Croatia. Only the initial diagnostic TUR biopsies from pT2 cases were analyzed, while repeat biopsies and patients with metastatic disease or other primary malignancies were excluded. Biopsies with insufficient tumor tissue for immunohistochemical analysis were also excluded. Patient data were obtained from hospital electronic medical records and included age, sex, and primary therapy, which was categorized as either conservative (repeat TUR with or without radiotherapy) or aggressive (cystectomy with adjuvant or neoadjuvant chemotherapy). Overall survival data were obtained from the Croatian National Cancer Registry.

### Pathological analysis

All biopsy slides were reviewed by a pathologist with an interest in uropathology (RT). Cases were histologically classified into subtypes according to the WHO 2022 classification [29] if more than 50% of the muscle-invasive tumor showed divergent morphology. The most representative block containing bona fide muscle-invasive tumor was selected for immunohistochemical analysis. All samples were fixed in 10% neutral buffered formalin for 24–72 h and embedded in paraffin. Two- to three-micron thick sections from all cases were stained with antibodies targeting GATA 3 transcription factor (GATA3), cytokeratins 5 and 6 (CK5/6), and p16 using appropriate external quality assurance methods. The combination of GATA3 and CK5/6 IHC has been shown to be a strong classifier of broad luminal (GATA3 positive, CK5/6 negative, or basal pattern staining) and basal (CK5/6 diffusely positive) subgroups in multiple previous studies [30–32]. Within the luminal group, consistently high expression level of p16 was found in the genomically unstable group according to the Lund classification system [33, 34]. The technical aspects of IHC are summarized in Table 1.

**Figure 1.**
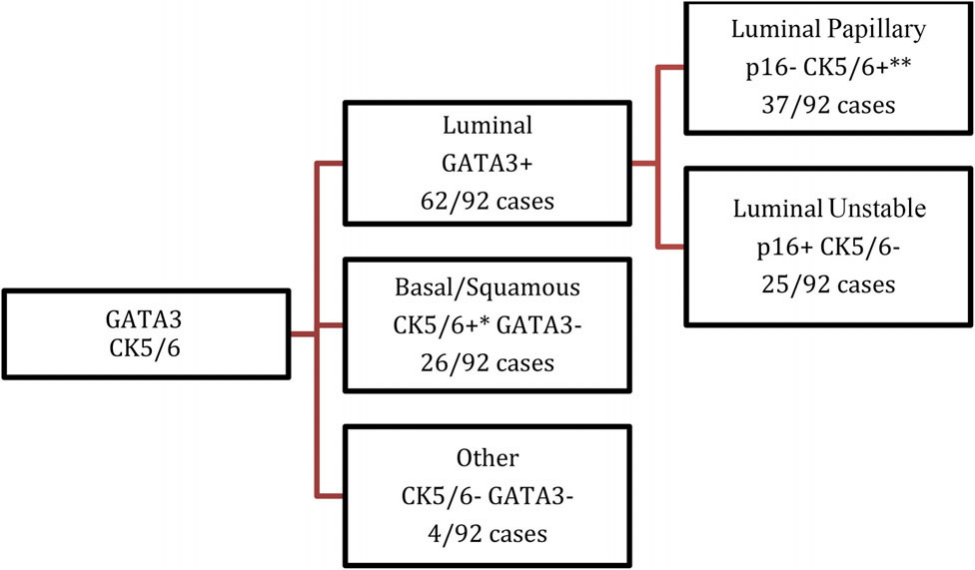
Algorithm used for assigning surrogate molecular subtypes based on immunohistochemical analysis (*diffuse staining pattern, **basal staining pattern). CK5/6: Cytokeratins 5 and 6.

**Figure 2.**
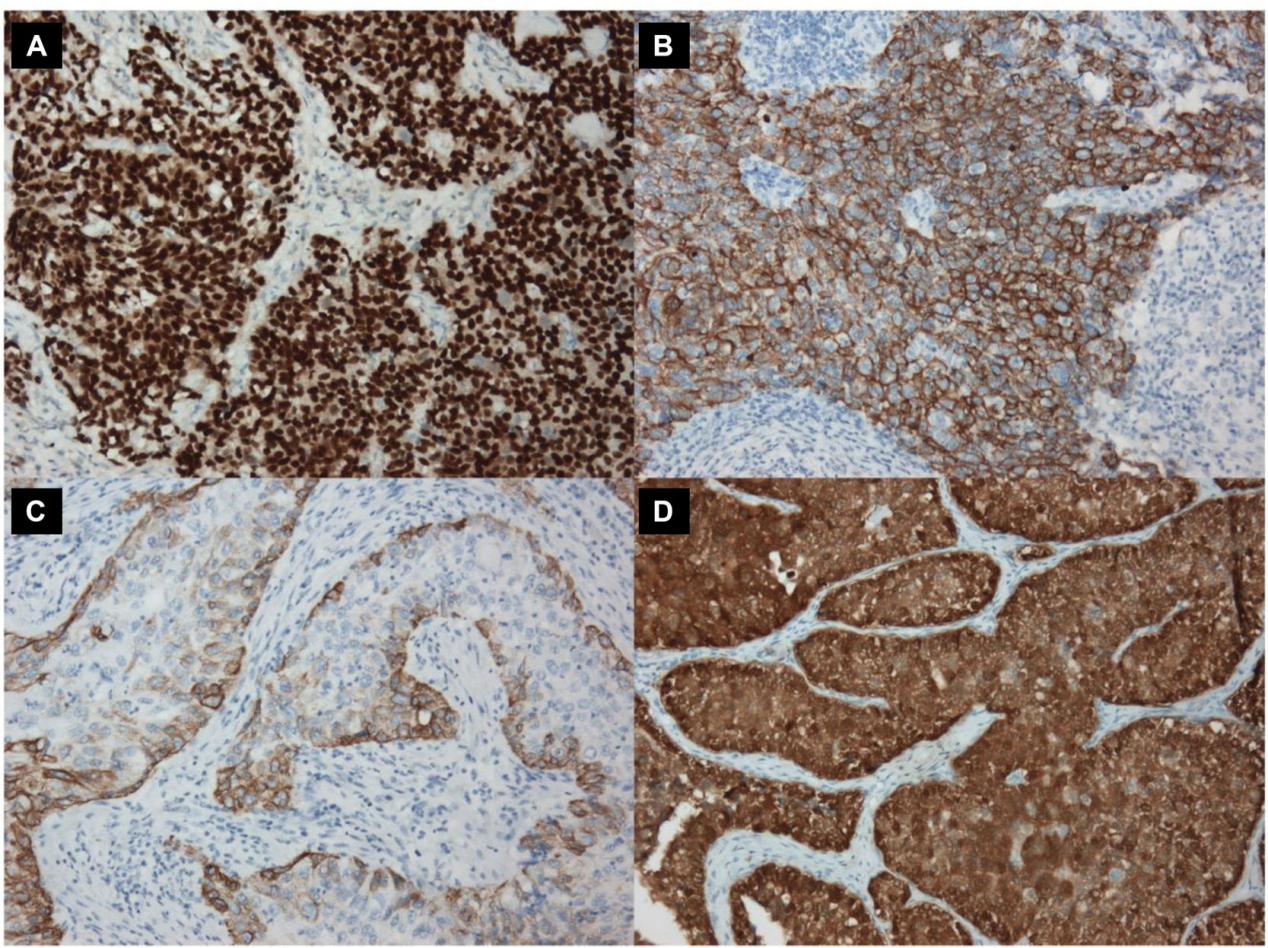
**Immunohistochemistry of** **muscle-invasive** **bladder cancer. **(A) Luminal Papillary type strong and diffuse GATA3 staining; (B) Basal/Squamous type strong diffuse CK5/6 staining; (C) Luminal Papillary type CK5/6 basal staining pattern; (D) Luminal Unstable type strong diffuse p16 staining; magnification 150×. CK5/6: Cytokeratins 5 and 6.

Scoring of IHC was performed only in the muscle-invasive part of the tumor. Based on the IHC scores, cases were assigned to a molecular surrogate class as shown in Figure 1. For GATA3 and CK5/6 staining, according to previously published recommended cutoffs [30], staining in more than 20% of tumor cells was considered relevant and scored as 2 (Figure 2A and 2B), staining in less than 20% was scored as 1 and negative staining was scored 0. Additionally, to be classified as score 2, the intensity of staining had to be high. The basal pattern of CK5/6 found in a proportion of luminal tumors [33] was scored as 1 (Figure 2C). For p16, strong diffuse staining in 70% or more of muscle-invasive tumor was considered score 2 (Figure 2D), less than 70% was scored as 1, while negative staining was scored as 0. For classification purposes, only cases with score 2 were deemed positive.

### Ethical statement

Institutional Ethics Board approval was obtained from the Clinical Hospital Center Sestre milosrdnice and the University of Zagreb School of Medicine, reference code 380-59-10106-21-111/211.

### Statistical analysis

Differences between categorical variables were calculated using the χ^2^ test. Survival curves were calculated using the Kaplan–Meier analysis, and non-adjusted differences between groups were tested for significance using the log-rank test. Univariate and multivariate hazard ratios were calculated with 95% confidence intervals using the Cox proportional hazards model. The significance level for all analyses was set at *P* < 0.05. All statistical analyses were performed using R (version 4.1.3) [35].

## Results

Ninety-two patients were analyzed. The mean age of the patients was 69.6 ± 9 years (range: 48–88 years), and 73% were men. Tumor volume was estimated from the number of blocks submitted to pathology. The mean was four blocks per patient ± 2.7 (range: 1–13 blocks). All cases were high-grade urothelial carcinomas, with 78 (84%) showing pure urothelial morphology and 15 (16%) showing divergent differentiation. Of these, 9 cases (10%) showed keratinizing squamous morphology, 2 (2%) plasmacytoid, 2 (2%) nested, 1 (1%) glandular, and 1 (1%) micropapillary morphology. Immunohistochemical analysis subclassified cases into LumP (37 cases, 40%), LumU (25 cases, 27%), and Ba/Sq (26 cases, 28%) molecular subtypes. In four cases (4%), double-negative staining was observed for both GATA3 and CK5/6. Patient characteristics by molecular subtype are summarized in Table 2.

**Table 2:** Patient characteristics according to the molecular subtype

	**Ba/Sq, *n* ═ 26**	**LumP, *n* ═ 37**	**LumU, *n* ═ 25**	**DN, *n* ═ 4**	**Statistics**
*Sex, n*					χ^2^ ═ 9.7
Female Male	13 13	7 30	4 21	1 3	df ═ 3 *P* ═ 0.02
*Age, years*					χ^2^ ═ 2.4
<70 ≥70	16 10	18 19	10 15	2 2	df ═ 3 *P* ═ 0.49
*Therapy, n*					χ^2^ ═ 2.4
TUR Cystectomy	12 14	24 13	13 12	2 2	df ═ 3 *P* ═ 0.49

Ba/Sq: Basal/Squamous; LumP: Luminal Papillary; LumU: Luminal Unstable; DN: Double negative; TUR: Transurethral resection.

Histologically, 78 cases showed pure urothelial morphology (of which 35 were LumP, 27 were LumU, 18 were Ba/Sq, and 4 were double negative by molecular immunophenotype), 9 cases showed squamous morphology with keratinization (all were Ba/Sq), 2 cases showed plasmacytoid morphology (1 LumP and 1 LumU), 2 cases showed nested morphology (1 Ba/Sq and 1 LumP), 1 case showed glandular morphology (LumU), and 1 case showed micropapillary morphology (LumU). No neuroendocrine or sarcomatoid morphologies were observed in our series.

Regarding therapy, 51 patients (55%) underwent conservative treatment (TUR with radiation), while 41 (45%) were treated with cystectomy and adjuvant or neoadjuvant chemotherapy.

**Figure 3.**
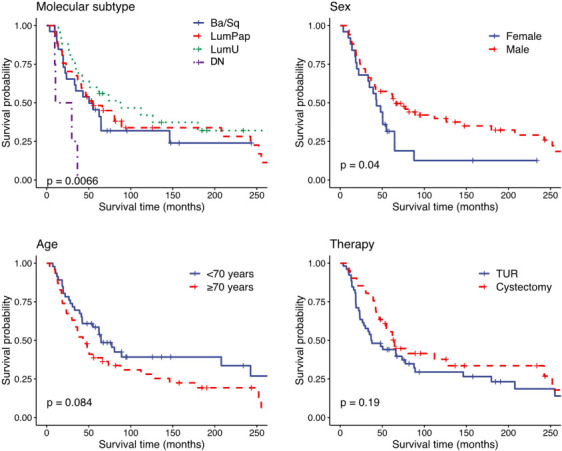
**Kaplan–Meier survival curves for sex, therapy, age, and molecular subtype (log-rank *P*** **on overall survival data).** TUR: Transurethral resection; Ba/Sq: Basal/Squamous; LumPap: Luminal Papillary; LumU: Luminal Unstable; DN: Double negative.

In univariate survival analysis, a statistically significant difference in overall survival was observed for sex and molecular subgroup (Figure 3), with women (*P* ═ 0.04) and double-negative cases (*P* ═ 0.007) having worse outcomes.

When adjusting for covariates using the Cox multivariate analysis, using the double-negative cases as reference, the differences for sex (HR 0.54, 95% CI 0.29–0.99; *P* < 0.05) and molecular subtype were confirmed for Ba/Sq (HR 0.19, 95% CI 0.04–0.83; *P* < 0.05), LumP (HR 0.15, 95% CI 0.04–0.57; *P* < 0.01) and LumU (HR 0.09, 95% CI 0.02–0.36; *P* < 0.001) subtypes (Table 3). No impact on survival was observed for age and therapy.

**Table 3:** Summary of univariate and multivariate survival analysis

			**Univariate analysis**		**Multivariate analysis**	
**Variable**	***N* ═ 92 number (%)**	**Overall survival months (median)**	**Unadjusted HR (95% CI)**	***P* value (log-rank test)**	**Adjusted HR (95% CI)**	***P* value (Wald test)**
*Age, years*						
≥70 (reference)	46 (50)	45				
<70	46 (50)	62	0.65 (0.40–1.06)	0.09	0.62 (0.35–1.09)	0.09
*Sex*						
Female (reference)	25 (27)	43				
Male	67 (73)	62	0.58 (0.33–0.99)	<0.05	0.54 (0.29–0.99)	<0.05
*Therapy*						
TUR (reference)	52 (56)	37				
Cystectomy	41 (44)	62	0.72 (0.44–1.18)	0.19	0.80 (0.46–1.38)	0.42
*Histology*						
Pure urothelialcarcinoma (reference)	78 (84)	48				
Squamous urothelialcarcinoma	9 (10)	63	0.80 (0.34–1.86)	0.60	0.41 (0.14–1.17)	0.10
Other histology	6 (6)	142	0.56 (0.20–1.56)	0.27	0.5 (0.17–1.51)	0.22
*Number of blocks*						
≤2 (reference)	77 (83)	48				
>2	16 (17)	56	0.93 (0.47–1.82)	0.83	0.50 (0.21–1.16)	0.10
*Molecular subtype*						
Double-negative(reference)	4 (4)	20				
Basal/Squamous	26 (28)	49	0.25 (0.08–0.76)	<0.05	0.19 (0.04–0.83)	<0.05
Luminal Papillary	37 (40)	48	0.22 (0.07–0.65)	<0.01	0.15 (0.04–0.57)	<0.01
Luminal Unstable	25 (27)	66	0.16 (0.05–0.49)	<0.01	0.09 (0.02–0.36)	<0.001

TUR: Transurethral resection*;* HR: Hazard ratio; CI: Confidence interval.

## Discussion

In modern precision oncology, MIBC has yet to reap the benefits of molecular subtyping approaches [5]. While mRNA-based molecular classification schemes have identified six subtypes of MIBC, these techniques are currently not feasible in routine practice due to high cost and associated complexity [36]. Moreover, these classification schemes were developed on tissue micro-array or fresh tissue samples, thus not accounting for potential tumor heterogeneity or formalin fixation effects [14–18, 20].

Different molecular subtypes could be associated with differences in response to specific treatment modalities. Therefore, the development of a useful, reliable, and cost-effective classification scheme would be of great benefit for regular patient care [10, 11, 26]. Similar to the well-established approach in breast cancer subtyping, IHC has the potential to be the method of choice in MIBC subtyping. Previously published IHC classification schemes have thus far used many antibodies, which are often not in routine clinical use [15, 33, 37–39].

The present study aimed to develop a simple and easily reproducible IHC method for subtyping the three major subtypes of urothelial carcinoma and to test it for prognostic relevance in a retrospective series. The method involves pathological review of whole slides with identification of the slide containing the most appropriate muscle-invasive focus on which to perform IHC. Immunohistochemical expression of GATA3 and CK5/6 has been shown to reliably classify cases into broad basal and luminal subtypes [30, 31]. In agreement with data from the University of Lund classification, we chose to add p16 as an economical way of further subclassifying the luminal type into LumP and LumU types [34]. In this way, using only three commonly available antibodies, we were able to assign 88/92 (96%) cases from our cohort to one of the three groups, namely, the LumP, LumU, and Ba/Sq subtypes, which represent the major subtypes of MIBC [37]. The respective proportions of all three groups were within the ranges of previously published molecular data (Table 4).

**Table 4:** Proportion of cases assigned to each molecular subtype compared with the published data

**Subgroup**	**% in current study**	**Published range**
Luminal Papillary	40	32%–46% [15, 17, 20, 46]
Luminal Unstable	27	15%–38% [20, 34, 38, 47]
Basal/Squamous	28	10%–45% [20, 38, 44, 46, 48]

While heterogeneity in invasive bladder cancer (i.e., cancer that has breached the basal membrane) is well known, especially in the Ba/Sq subtype, it is not entirely clear from previous studies, some of which used tissue microarray approaches, whether the tissue analyzed consisted only of muscle-invasive foci or also included in situ or T1 disease [34, 37, 38, 40–42]. In our study, all muscle-invasive foci did not show more than one histologic and immunohistochemical phenotype, suggesting that this approach could be viable when subtyping cases with limited muscle-invasive areas, such as TUR specimens, while heterogeneity could best be investigated in cystectomy specimens.

In the study by Makboul et al. [38], an immunohistochemical subtyping method was developed using antibodies against fibroblast growth factor receptor 3 (FGFR3), human epidermal growth factor receptor 2 (HER2), p53 protein, cyclin B1 (CCNB1), and CK5 to subclassify both nonmuscle-invasive urothelial carcinoma and MIBC. Subtypes were assigned using the initial Lund scheme. Even though this approach is more complex and could not successfully classify 7.8% of cases, one advantage is the upfront analysis of potential therapeutic targets in the form of anti-FGFR and anti-HER2 therapy.

Goutas et al. [43] analyzed the expression of GATA3 and CK5/6 in 77 Ta, T1, and T2 bladder carcinomas and classified the cases into a luminal (GATA3+/CK5/6−) and a basal (GATA3−/CK5/6+) subtypes. Additionally, they analyzed PD-L1 expression and found a strong correlation with basal subtype, in line with previous data.

In the recently published study by Olkhov-Mitsel et al. [44], the authors used the same three-antibody classifier as in the present study on tissue microarray material from chemotherapy-naive cystectomy specimens. In their series, 97.1% of MIBC cases were classified as either luminal GU, luminal Uro, or basal according to the Lund scheme. To explain their findings in terms of consensus classification, the authors proposed to correlate the luminal GU type to the LumU group, the luminal Uro to the LumP and LumNS, and the basal group to the Ba/Sq and stroma-rich groups. The remaining GATA3 and CK5 negative cases were hypothesized to belong to the neuroendocrine-like group.

In the study by Queipo et al. [39], immunohistochemical subtyping was performed according to the Lund classification scheme, and cases that were GATA3 and CK5/6 negative were assigned to the Mesenchymal-like and Neuroendocrine groups based on the presence or absence of sarcomatoid or neuroendocrine features histologically [39]. According to the consensus classification by Kamoun et al. [20], most Lund Mesenchymal-like cases belong to the stroma-rich subtype, while some belong to the Ba/Sq type. Additionally, the consensus classification of LumNS group includes cases of several (mostly Luminal) Lund subtypes. Queipo et al. also described 2 of 113 cases initially considered as GATA3, CK5/6, and p16 negative which upon review showed weak CK14 and CK5/6 positivity and were then classified as Luminal and Uro based on patchy and parabasal CK5/6 pattern, respectively.

In our study, four cases showed a GATA3 and CK5/6 negative phenotype with a positive p16. This double-negative group showed worse overall survival compared with LumU, LumP, and Ba/Sq cases. Given that in our series, these cases showed urothelial morphology, they might be tentatively classified as LumNS cases.

A recent study by Bejrananda et al. [45] assessed GATA3 and CK5/6 IHC on tissue microarray material of 132 radical cistectomy cases and classified tumors into four groups (Luminal, Basal, Double negative, and Mixed). Their data showed improved overall survival in patients with mixed (GATA3 and CK5/6 positive) expression, while overall survival was not significantly different in the other groups.

Future immunohistochemical subtyping studies should continue to combine morphologic and immunohistochemical parameters, thus maintaining the central role of the practicing pathologist in subclassification efforts. In this way, a cost-effective approach to the subclassification of MIBC could be developed in accordance with consensus classification.

Our study had several limitations. First, this study was prone to selection bias and other limitations pertinent to the retrospective single-cohort design. Second, while efforts on molecular classification of bladder cancer are relevant for future research, we acknowledge that treatment decisions based on molecular subtypes are not currently made because data are conflicting [36] and mostly limited to retrospective series. Third, we chose overall survival as an endpoint, although we had a small sample with a limited number of events. For future studies, we plan to explore other relevant endpoints, such as disease-free survival and local relapse as these endpoints, besides being enriched with more events, are potentially more clinically meaningful for practicing urologists and oncologists.

## Conclusion

A simple IHC panel using three routine antibodies is a promising tool for molecular subtyping of MIBC. In the present study, a difference in overall survival was observed between different molecular subtypes. To validate the classification scheme, multicentric prospective trials involving modern therapeutic approaches, such as immunotherapy with a large sample size, are needed.
